# Factors associated with the occurrence and level of *Isospora suis* oocyst excretion in nursing piglets of Greek farrow-to-finish herds

**DOI:** 10.1186/1746-6148-8-228

**Published:** 2012-11-22

**Authors:** Vasilis Skampardonis, Smaragda Sotiraki, Polychronis Kostoulas, Leonidas Leontides

**Affiliations:** 1 Laboratory of Epidemiology, Biostatistics and Animal Health Economics, University of Thessaly, 224 Trikalon st, 43100, Karditsa, Greece; 2 Veterinary Research Institute, National Agricultural Research Foundation, Nagref Campus, PO Box 60272, 57001, Thermi, Greece

**Keywords:** *Isospora suis*, Oocyst excretion, Risk factor, Two-part model

## Abstract

**Background:**

Piglet isosporosis is one of the most common parasitic diseases in modern pig production. To prevent clinical disease, prophylactic treatment of piglets with toltrazuril (BAYCOX® 5%, Bayer HealthCare, Animal Health, Monheim, Germany) is widely practiced in the past 20 years. There are only very few reports documenting the likely effect of managerial practices, such as hygiene measures, all-in-all-out management of farrowing facilities and piglet manipulations, and/or farm-specific environment - i.e. design and materials of the farrowing pen and room - in the risk of disease occurrence and transmission. Therefore, in this cross-sectional study, we identified litter- and herd-level factors associated with the odds and the level of *Isospora suis* oocyst excretion in nursing piglets of Greek farrow-to-finish pig herds. Faecal samples were collected from 314 liters of 55 randomly selected herds. Oocyst counts were determined by a modified McMaster technique and possible risk-factor data were collected through a questionnaire. In the analysis, we employed a two-part model that simultaneously assessed the odds and the level of oocyst excretion.

**Results:**

Factors associated with lower odds of oocyst excretion were: use of toltrazuril treatment, all-in all-out management of the farrowing rooms, no cross-fostering or fostering during the first 24 hours after farrowing, plastic flooring in the farrowing pens, farrowing rooms with more than fourteen farrowing pens and employment of more than two caretakers in the farrowing section. Factors associated with lower oocyst excretion level were: use of toltrazuril treatment and caretakers averting from entering into farrowing pens.

**Conclusion:**

Apart from prophylactic treatment with toltrazuril, the risk and the level of *I*. *suis* oocyst excretion from piglets in their second week of life, was associated with managerial and environmental factors. Changes in these factors, which may enhance prevention of piglet isosporosis – either alternatively or supplementary to medical control – are of increasing importance because of the likely development of resistant parasites under the currently widespread use of anticoccidial compounds.

## Background

*Isospora suis* is one of the most prevalent parasites in intensive pig production worldwide [1,2] and can cause significant economic losses due to transient diarrhoea and dehydration in nursing piglets followed by decreased weight gain and poor performance [3,4]. The disease occurs mainly in the second to third week of life and is characterised by high morbidity and low mortality except for cases with secondary bacterial infections [5,6]. Once *I*. *suis* has been established in a farm, the infection is probably maintained through transmission from one generation of piglets to the next [7] via contaminated farrowing pens [8,9]. Sows are rarely found to excrete oocysts [9], and may not play a critical role for *I*. *suis* infection persistence in an infected herd [2].

*I*. *suis* is present in all types of farrowing facilities and under all types of management systems [10], regardless of herd size and housing conditions [6,11]. A study carried out in 12 European countries confirmed the presence of *I*. *suis* in 26% of litters and in 69% of the herds examined [12]. Studies conducted in several European countries estimated the prevalence of clinical isosporosis and identified factors – like hygiene measures and perforated pen floors – which appeared to lower the risk of disease [1,13].

Several aspects of the *I*. *suis* epidemiology have not been fully conceptualized yet. There are only very few reports documenting the likely effect of managerial practices, such as hygiene measures, all-in-all-out management of farrowing facilities and piglet manipulations, and/or farm-specific environment - i.e. design and materials of the farrowing pen and room - in the risk of disease occurrence and transmission [1,14,15]. Unveiling the epidemiology of the infection is a prerequisite for the control of the disease through interventions in management procedures [2]. This explicitly falls within the concept of the “component cause”: the identification of the specific mixture of necessary conditions and events (i.e. duration and level of exposure to the pathogen and the presence of animal- and herd-specific risk factors) which are both necessary and sufficient to produce *I*.*suis* infection and determine the future course of the infection [16]. Factors varying at the litter-level – such as the age at initial infection, infection dose, duration of exposure and the age-related resistance to re-infection [2,17-20] – may affect the relation between the oocyst excretion pattern and clinical isosporosis. Interestingly, even under identical animal housing conditions and experimental infection procedures considerable within and between litter variation [19,20] in oocyst excretion levels and diarrhoea occurrence [6] has been observed. Currently, no field studies aiming to identify factors associated with the risk and/or the level of oocyst excretion have been reported. Factors operating at the litter- or herd-level may account for a significant portion of the among-herds variation in the risk of occurrence of oocyst excretion, the level of excretion and the risk of diarrhoea [21]. In this study we aimed at quantifying the effect of litter- and herd-level factors on the odds of occurrence and the level of *I*. *suis* oocyst excretion in nursing piglets of Greek farrow-to-finish pig herds.

## Methods

### Pilot study

A pilot study, including ten herds, randomly selected from the country’s national registry, was conducted before the initiation of the study, to obtain prior information for estimation of the required sample sizes. From each of these ten herds, ten randomly selected litters in their second week of life were sampled and tested as described below (section ‘Parasitological methods’). Based on the data collected from the pilot study, we estimated an intra-herd correlation coefficient (ICC) for oocyst excretion of 0.4, and detected excretion in 20% and 80% of the litters in herds treated (six herds) or not (four herds) with toltrazuril (BAYCOX® 5%, Bayer HealthCare, Animal Health, Monheim, Germany), respectively.

### Sample size determination

Based on the results of the pilot study a minimum difference of 60% was expected in the proportion of positive litters between those treated and not treated with toltrazuril. The minimum required sample size for comparing two proportions, based on the standard sampling formulae, equals to 26 litters, 13 for each group. Sample size calculations were done with the Piface java applet [22]. However, in the presence of clustering, standard individual-based sample size formulae do not account for the between herd variation [23], and therefore, estimated sample sizes must be inflated by the variance inflation factor: VIF = 1 + ICC*(*ms*-1) [24], where *ms* is the mean number of litters sampled in each herd. The ICC under the pilot study was 0.4 and assuming an *ms* = 5, VIF was equal to 2.6. Thus, a total of 26*2.6 = 68 litters was required. In order to increase the statistical power for identifying risk/preventive factors which may be strongly correlated with toltrazuril treatment, an almost 5-fold increase in the total sample size, which could be financially supported by the study’s budget, was decided. Thus, a total of 345 litters from 60 farrow-to-finish herds were sampled.

A two-stage sampling design was used. Initially, herds were randomly selected from the country’s national registry, after excluding herds with less than 20 sows and those located on the Greek islands. Consent for participation in the study was obtained after personal communication of the owner or the manager of the farm with the primary author. When they denied participation, the farm located closest was contacted and, if agreed to participate, was sampled. Subsequently, litters were randomly sampled within the selected herds.

### Collection of questionnaire data

To collect data on factors likely affecting the risk of oocyst excretion we developed a questionnaire (see Additional file 1) which, based on previous reports [25,26], aimed to detect two potential clusters of risk/preventive factors relating to (i) hygiene practices and (ii) characteristics of the farrowing facilities. Eighty questions were included on factors that varied either at the litter- or herd-level. For herd-level factors all animals/litters in the same herd will have the same characteristic, whereas, litter-level factors are independent of herd and can vary between litters of the same herd [27].

The data included information on herd size, production parameters, housing conditions, managerial strategies, disease prevention, hygiene practices, cleaning and disinfection procedures, farrowing room and pen design as well as application of toltrazuril treatment. Fifty-three questions were closed (e.g. yes/no, always/frequently/seldom/never or pre-set options), twenty-one were semi-closed (e.g. information on number of days, farrowing pens and rooms, application frequencies of certain procedures) and the remaining were open-ended (e.g. product names, descriptions). The interviewer checked the accuracy of some data, such as size and location or flooring of the farrowing pen, by inspecting the farrowing facilities. Questionnaires were filled-in, before sampling, with personal interview of either the owner or manager of the herd by the first author (VS). The interviewer had no prior knowledge of the *I*. *suis* status of the herds.

### Parasitological methods

From each of the selected herds five to ten litters in their second week of life (from day 8 to 14 post farrowing (p.f.)) were sampled. The cross-sectional type of the study and the fact that all the studied farms were managed with continuous farrowing, justified the variable number of sampled litters. Evidently, at the herd visit we could find more litters in the second week of life in larger than in smaller herds. Each sampled litter represented a different number of litters in small than in large herds. Faecal samples were collected, using a swab, from the rectum [2] of each from half of the piglets of each litter and then pooled. At least two grams of faeces were collected from each sampled litter [1]. Each pooled sample was stored individually in labelled plastic containers. To avoid cross-contamination, plastic protective shoes and gloves, which were changed between litters, were used during sampling. Oocyst concentration was determined by a modified McMaster technique using saturated sodium chloride solution with 500 g glucose per litter, as flotation fluid [13,28]. The oocysts were counted in glass McMaster chambers under a fluorescence light source using UV excitation (340–380 nm) and oocyst excretion was expressed as oocysts per gram of faeces (OPG) [2,29]. The method has a lower detection limit of 20 OPG. Piglets of infected litters usually acquire *I*.*suis* early in their life. It is conceivable that few (one or two) lately infected piglets might give such a low non-detectable value but the outcome from an infected litter pool would definitely be positive. Thus, the probability that an infected litter is misclassified as negative is minimal when a pool of faeces from half of the piglets of each litter is tested.

Each pooled faecal sample was considered positive for oocyst excretion if the minimum concentration of 20 OPG was detected and the respective litter was classified as one that excreted oocysts. When all litters from a herd were negative, we used a standard protocol of re-sampling the same litters one week later to minimize the probability of misclassifying an infected herd [11,13]. Only, the results obtained at re-sampling were used in the analysis. A herd was considered positive if at least one litter sample was positive.

### Statistical analysis

#### Assessing the odds and the level of oocyst excretion

**Figure 1:**
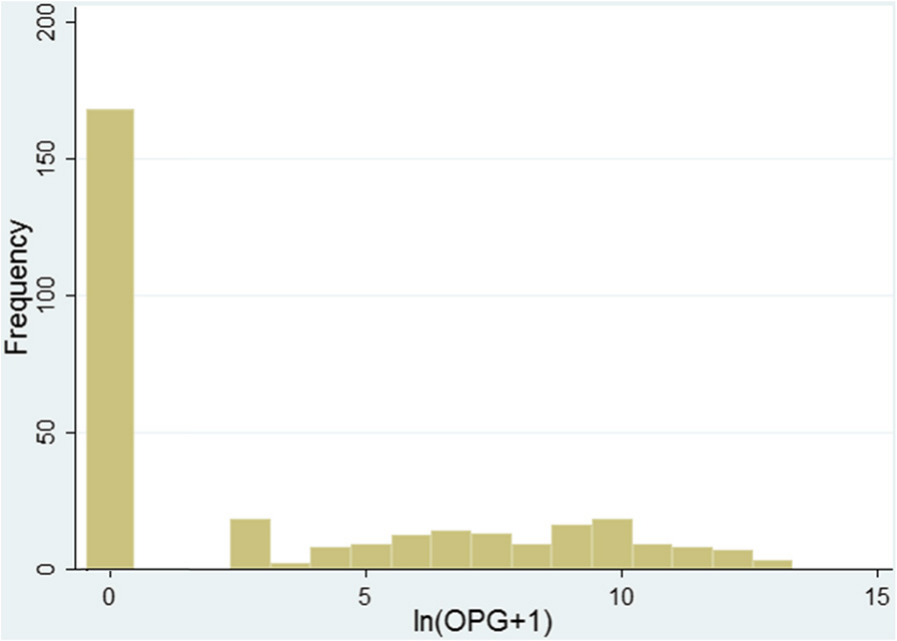
**Frequency distribution of the natural logarithm of *I*.*suis *oocysts per gram of faeces[ln(OPG + 1)]from pooled faecal samples of 314 litters in 55 herds.** Faecal samples were collected from half of the piglets of each litter and then pooled, during their second week of life. OPG counts were determined by a modified McMaster technique.

OPG counts were semi continuous data (Figure 1), characterized by the presence of a large portion of zero values and skewing to the right of the non-zero values [30]. Logarithmic transformation can be used to normalize the positively skewed non-zero values but excess zeros remain a problem. Using a logistic regression, after recording data into a dichotomy (zeros vs. ones) is a common approach, but important information is discarded since the question of “how much” something occurred is reduced to “whether or not” it occurred [30]. Two-part models can account for the concentration of excessive zero-valued observations. This approach uses logistic regression to predict the probability of occurrence of a non-zero value in the first part, and linear regression to predict the amount of the non-zero values in the second part [31].

We employed a two-part model with the presence or absence of oocyst excretion at the litter-level as the response variable in the first part of the model, while the natural logarithm of the non-zero OPG was the response variable in the second part. Random-effect terms were incorporated in the model to account for the within herd correlation of observations for both the odds and level of oocyst excretion. Furthermore, to capture the biologically plausible fact that herds with higher rates of oocyst excretion may also have higher OPG counts, we adopted a structure that adjusted for the cross-equation correlation of the random-effects terms between the logistic and linear part of the model [31]. Lastly, sampling weights were used in order to adjust for the unequal selection probabilities of litters originating from herds of unequal size. Specifically, these weights denoted the inverse of the probability that each litter from each herd is included because of the sampling design, thus, giving increased weights to litters of larger herds.

For model building, all candidate variables were initially screened, one-by-one, using a bi-variable approach [32]. Toltrazuril treatment was forced into both parts of all models because it is known to reduce both the odds and the level of excreted oocysts [21]. For the past fifteen years, prophylactic toltrazuril treatment was inherent to the control of swine isosporosis in Greece. Thus, the assessment of the effect of the candidate variables on the risk and the level of OPG excretion should adjust for the use of toltrazuril treatment. During this screening phase, a significance level of 0.25 was used [33]. Then, variables with *P* < 0.25 in both or either part were simultaneously offered to a full model which was, subsequently, reduced by backwards elimination [34], until only significant (*P* < 0.05) variables remained. When pairs of highly correlated variables were encountered, selection of the variable to be included in the model was based on biological plausibility. Two-factor interactions were created between the remaining variables and offered one at a time to the model. Finally, a stepwise forward selection process was done by offering previously excluded variables to the final model one at a time.

#### Goodness of fit

To check the adequate fit of the model to the observed data, we simulated the infection status (oocyst excretion or not) and the amount of oocysts excreted for each litter, under the final model. The simulated data under each part of the model were then compared to the observed data [31,35]. Briefly, we simulated 10,000 samples from each part of the model and formed the 100*q% equal tailed credible intervals. The model under consideration is adequate for the data if at least 100*q% of the actual observations lie in this interval. For the logistic part of the model, the agreement between the simulated and the actual oocyst excretion status was high. For the linear part more than 95% of the actual observations were within the 95% credible intervals of the simulated values, suggesting an adequate fit of the model.

#### Statistical software

Estimation of the two-part random effects model was performed using the freely available software aML (*aML Multilevel Multiprocess Statistical Software*: *Version 2*.*0*., Econ Ware: Los Angeles) which supports multilevel models and takes into account, by default, the different level of hierarchy of the fitted covariates [36].

## Results

All but one of the selected farmers consented to participate in the study. Data from five herds were not considered in the analyses because the farmers failed to adequately fill-in several questions. Thus, the data analysed comprised of fifty-five herds, totalling 314 sampled litters. Only two out of the ten herds which were initially found negative, were found positive at re-sampling while the remaining eight herds tested negative. From the herds found infected, 146 of the sampled litters tested positive (Table 1).

**Table 1:** **Proportion of positive litters and mean** (**SD**) **of the natural logarithm for the OPG of the positive litters by the factors selected for multivariable analysis**, **in a two**-**part model**, **under tri**-**variable screening** (**with factor TOLTRAZURIL forced in the model**), **for the association with the odds** (**logistic part** - **Part I**) **and the level** (**linear part** - **Part II**) **of *Isospora suis *oocyst excretion**

**Definition of factor**	**Level**	**Number of litters**	**Proportion of positive litters**	**Mean(SD)ln(OPG)**	***P***
**Part I** + **Part II**					
Use of toltrazuril	Yes	200	0.30	6.6 (3.1)	0.001^a^
	No	114	0.65	8.2 (2.7)	0.002^b^
Piglet age at weaning	*cont*.	-c	-	-	0.116^a^
					0.190^b^
Number of pens per farrowing room^*^	>14	154	0.31	7.0 (3.3)	0.035^a^
	≤14	160	0.52	7.6 (2.8)	0.067^b^
Remaining faeces after cleaning	Yes	88	0.52	8.0 (3.0)	0.056^a^
	No	226	0.37	7.1 (2.9)	0.145^b^
Size of farrowing pen in m^2*^	*cont*.	-	-	-	0.042^a^
					0.113^b^
**Part I**					
Cross-fostering of piglets	Within the 1st day p.f.^**^ no fostering	60	0.26	6.8 (2.5)	0.061
	After 1st day p.f.	254	0.44	7.4 (3.1)	
Cleaning entire farrowing room	Yes	193	0.34	7.4 (2.7)	0.077
	No	121	0.63	7.3 (3.5)	
Disinfection of farrowing unit on a regular basis	Yes	290	0.40	7.4 (3.0)	0.050
	No	24	0.56	7.6 (3.3)	
Flooring of farrowing pen in creep area^*^	Perforated	91	0.27	6.3 (3.1)	0.070
	Solid	223	0.51	7.8 (2.9)	
Number of caretakers in farrowing unit	>2	55	0.24	6.3 (2.7)	0.186
	≤2	259	0.48	7.6 (3.0)	
Farrowing pen flooring^*^	Plastic	89	0.38	7.1 (3.3)	0.160
	Metal	225	0.41	7.5 (2.9)	
Slatted portion of farrowing pen’s flooring^*^	30%	55	0.27	8.5 (2.3)	0.171
	60%	111	0.48	7.7 (2.6)	
	100%	148	0.40	6.7 (3.3)	
**Part II**					
Use of drying substances	Yes	115	0.31	7.3 (2.9)	0.170
	No	199	0.48	7.4 (3.1)	
Caretakers entering pens	Yes	294	0.41	7.5 (3.0)	0.110
	No	20	0.34	5.7 (2.4)	
Mechanical ventilation in farrowing unit	Yes	207	0.39	7.5 (2.6)	0.169
	No	107	0.45	7.0 (3.8)	
Washing water	Cold	255	0.40	7.4 (3.2)	0.002
	Hot	59	0.38	7.1 (2.5)	

Dual P-values correspond to factors that were offered to both parts of the model. Data are from 314 litters in 55 farrow-to-finish Greek pig herds.

^a^*P*-values for Part I. ^b^*P*-values for Part II. * Litter-level variables. ** Post farrowing.

After questionnaire data compilation, fifty-three variables (18 litter- and 35 herd-level) were initially screened as candidates for inclusion in the final two-part model. Those with *P* < 0.25 in the tri-variable analysis are in Table 1. After selection of the parsimonious multivariable model, none of the tested interaction terms was significant (P < 0.05). Also, none of the previously excluded variables was significant. The final two-part model included six factors in the logistic part and two factors in the linear part (Table 2).

**Table 2:** **Herd**- **and litter**-**level factors of the final multivariable two**-**part model**, **associated with the odds and the level of *Isospora suis *oocyst excretion**

**Part I**				
**Variable**	**Parameter**	**OR**	**95% C.I.**	***P***
Use of toltrazuril	Yes	1	-	
	No	3.7	1.6; 9.1	0.005
Clean entire farrowing room	Yes	1	-	
	No	3	1.0; 9.0	0.045
Cross-fostering of piglets	Within the 1st day p.f.^**^ or no fostering	1	-	
	After 1st day p.f.	4.2	1.2; 15.6	0.039
Farrowing pen flooring^*^	Plastic	1	-	
	Metal	2.8	1.2; 6.5	0.022
Number of pens per farrowing room^*^	>14	1	-	
	≤14	3.7	1.8; 7.7	0.001
Number of caretakers in farrowing unit	>2	1	-	
	≤2	2.7	1.3; 5.9	0.015
**Part II**				
**Variable**	**Parameter**	**Coeff.**	**95% C.I.**	***P***
Use of toltrazuril	Yes	−1.9	−0.8; -3.1	0.002
	No	0	-	
Caretakers entering pens	Yes	1.3	0.2; 2.5	0.024
	No	0	-	

* Litter-level variables. ** Post farrowing.

Litters in herds using toltrazuril treatment had 3.70 [95% Confidence Interval (CI): 1.56; 9.10] times lower odds of being positive compared to litters in herds not treating. Application of cleaning and disinfection procedures to the entire farrowing room, after all litters were weaned, compared to split-weaning of the room and cleaning only the pens of the weaned litters, decreased the odds of litter positivity by 3.00 (1.03; 9.00) times. Litters in which cross-fostering of piglets was performed after the first day p.f. had 4.20 (1.15; 15.64) times higher odds of positivity, compared to either not applying cross-fostering of piglets or performing this practice during the first 24 hours p.f. Litters in farrowing pens with metal perforated floor had 2.81 (1.19; 6.52) times higher odds of excreting oocysts, compared to litters in pens with plastic perforated floor. Litters in farrowing rooms with more than fourteen pens had 3.70 (1.81; 7.69) times decreased odds in excreting oocysts. Finally, in the herds where more than two caretakers were employed in the farrowing section, litters had 2.70 (1.28; 5.88) times lower odds of being identified as positive.

From the linear part, litters in herds using toltrazuril treatment had 7.03 (2.23; 22.20) times lower mean OPG compared to herds not using toltrazuril treatment. Further, litters in herds where caretakers of the farrowing section entered in the farrowing pens, in order to perform the necessary manipulations in piglets, had 3.85 (1.22; 11.94) times higher mean OPG compared to herds where caretakers avoided entering the farrowing pens.

The herd-level variance was 0.82 (standard error: 0.19) and 1.49 (0.33) for the logistic and the linear part, respectively. The cross equation correlation between the herd-level random effects of the logistic and the linear part was 0.95, revealing that herds at higher risk of excretion were more likely to also experience higher excretion levels (*P* < 0.008).

## Discussion

We performed this cross-sectional study in order to identify litter- and herd-level factors affecting the odds of occurrence and the level of *I*. *suis* oocyst excretion in nursing piglets of Greek farrow-to-finish herds. Alterations in managerial practices and environmental factors are likely to contribute to the control of isosporosis through the reduction rather than the elimination of oocyst excretion, at least in the medium term. Thus, from an epidemiological point of view, there is an interest in the identification of risk factors that reduce the risk and/or the level of oocyst excretion. The majority of the candidate risk/preventive factors, and those in the final model, can be considered constant over time since they represent either routine managerial practices or refer to properties of housing facilities. They were not subject to modification depending on the perceived or observed oocyst excretion risk and, hence, minimized the limitations arising from the cross-sectional design. This design, however, may not have captured the well-accepted daily variation in oocyst excretion levels. This may explain the fact that the majority of the factors identified in our analysis were associated with the odds and not the level of oocyst excretion (Table 2).

### Risk factors

Application of early routine treatment with toltrazuril reduced both the odds and the level of oocyst excretion. The efficacy of this treatment against piglet coccidiosis has been previously demonstrated in experimentally infected piglets [36,37] as well as under natural infection conditions [19,21,38,39]. Toltrazuril affects all endogenous parasite stages [40] and suppresses the development of oocysts [41]. Treatment delays the onset of oocyst excretion and decreases both the odds of oocyst excretion and the mean amount of excreted oocysts [21]. Therefore, it lowers infection pressure and contributes to a slower and incomplete spread of *I*. *suis*[40], until piglets are sufficiently resistant to both infection and clinical isosporosis [17,37].

Herds practising cross-fostering of piglets after day one p.f., had litters with higher odds of oocyst excretion compared to litters in herds where cross-fostering was not applied or done within the first 24 hours p.f. Late cross-fostered piglets may shed earlier and may be responsible for litter over-contamination. Early fostering reduces exposure of piglets to the stress of fostering. The severity and duration of diarrhoea are greater and earlier in cross-fostered piglets compared with resident counterparts despite that fact that the former excrete less oocysts [17,19]. The latter may be partly ascribed to the absence of a solid immunity status in late cross-fostered piglets [17], which are moved in an environment with pathogens against which they do not have adequate protection. Further, late cross-fostering could lead to the introduction of piglets from an infected litter to an uninfected one or one with low shedding.

Cleaning and disinfecting the entire farrowing room after all litters were weaned reduced the odds of oocyst excretion. The proposed approach is an indication of a relatively high standard in the practiced hygiene procedures in a farm, as part of an integrated all-in-all-out management. Reduced environmental contamination, by thorough cleaning, can be effective in preventing or delaying initial *I*. *suis* infections in very young suckling piglets [2] and farms applying above-average hygiene measures have decreased infection rates [13]. Cleaning the entire farrowing room at once, instead of cleaning the pen of each weaned litter, provides a simultaneous and homogeneous reduction of the contamination level in all pens. Complete removal of oocysts from the environment is practically unfeasible; however, lower infection pressure can reduce the odds of clinical disease [25], initiating a slower and incomplete spread of the disease [37].

We failed to identify a significant association of the perforated proportion of pen flooring with the odds or the level of oocyst excretion. However, a significant effect was observed for the type of perforated flooring: plastic flooring decreased the risk of oocyst excretion compared to metal flooring. Likely, this is because plastic flooring is a material that can be easily and effectively cleaned, thus, leading to a reduced number of ingested oocysts. Metal flooring, compared with plastic, is harder to maintain clean [42], thus the applied sanitary procedures could more effectively remove infectious oocysts in farrowing pens with plastic flooring, compared to metal flooring, resulting in decreased infection pressure for the successive litter.

Litters in farrowing rooms with large number of farrowing pens had decreased odds of occurrence of oocyst excretion. An important route of *I*. *suis* transmission, besides the one between successive litters (one generation of piglets to the next), is from one oocyst excreting litter to a neighbouring one, via mechanical carriers [26]. The aforementioned between-pen transmission and eventually built-up of the environmental contamination may be favoured in smaller than larger farrowing rooms. Smaller rooms may be looked after by only one caretaker while this may be less likely in larger units. Room size was correlated (*P* = 0.03) with the number of caretakers employed in that section. Furthermore, the size of the farrowing room may be considered as a proxy variable for a mixture of good managerial practices which are more consistently and efficiently adhered to in larger farms.

The presence of more than two caretakers in the farrowing section resulted in decreased odds of oocyst excretion. The existence of more caretakers could provide adequate manpower to maintain a relatively constant and high level of hygiene. Moreover, hygiene is sometimes neglected during work-intensive periods [13], thus more caretakers, regardless of herd size, can ensure a higher ratio of labour dedicated to cleaning. In some herds all routine manipulations in piglets by caretakers were carried out without them entering in the farrowing pens. Those litters had a lower infection level, as expressed by lower oocyst excretion. This practice has been proposed as an additional preventive measure for control of isosporosis. Limited access of farm workers to pens, with possibly infected piglets, can prevent pen-to-pen spread of infection via mechanical transfer of oocysts with boots [9]. Furthermore, others [26] suggested that the most important route of transmission is not from one litter to the next within the same pen, but more likely from one oocyst excreting litter of piglets to a neighbouring litter of younger and highly susceptible piglets, for example, via contaminated boots of the animal caretaker.

## Conclusions

We have assessed the impact of several managerial factors on the odds and the level of *I*. *suis* oocyst excretion. The role of and the need for identification of such interventions which could be used as preventing measures against isosporosis – either alternatively or supplementary to medical control [2,26,43] – are of increasing importance because of the likely development of resistant parasites under the currently widespread use of anticoccidial compounds [44].

## Abbreviations

(ICC): Intra-herd correlation coefficient; (VIF): Variance inflation factor; (p.f.): Post farrowing; (OPG): Oocysts per gram of faeces.

## Competing interests

The authors declare that they have no competing interests.

## Authors’ contributions

VS carried out the collection of the samples, parasitological testing, statistical analysis and drafted the manuscript. SS participated in the design of the study, the parasitological testing of the samples and the drafting of the manuscript. PK participated in the design of the study, the development of the statistical models and the drafting of the manuscript. LL conceived and coordinated the carry-out of the study and was involved in the statistical analysis, the drafting and the critical review of the manuscript. All authors have read and approved the final manuscript.

## Supplementary Material

Additional file 1 Piglet isosporosis questionnaire. Click here for file

## References

[B1] Mundt HC Cohnen A Daugschies A Joachim A Prosl H Schmaschke R Westphal B Occurrence of isospora suis in Germany, Switzerland and Austria J Vet Med B 2005 52 93 97 10.1111/j.1439-0450.2005.00824.x 15752269

[B2] Sotiraki S Roepstorff A Nielsen JP Maddox-Hyttel C Enoe C Boes J Murrell KD Thamsborg SM Population dynamics and intra-litter transmission patterns of Isospora suis in suckling piglets under on-farm conditions Parasitology 2008 135 395 405 18021464 10.1017/S0031182007003952

[B3] Stuart BP Lindsay DS Ernst JV Gosser HS Isospora suis enteritis in piglets Vet Path 1980 17 94 93 7352366 10.1177/030098588001700109

[B4] Stuart BP Lindsay DS Coccidiosis in swine Vet Clin N Am-Food A 1986 2 455 468 10.1016/s0749-0720(15)31256-1 3488114

[B5] Stuart BP Lindsay DS Ernst JV Acres SD Coccidiosis as a cause of scours in baby pigs Proceedings of the Second International Symposium on Neonatal Diarrhea, Veterinary Infectious Disease Organization, Saskatchewan, Canada 1978 Canada: University of Saskatchewan 371 382

[B6] Lindsay DS Blagburn BL Powe TA Enteric coccidial infections and coccidiosis in swine Comp Cont Educ Pract 1992 14 698 702

[B7] Eysker M Boerdam GA Hollanders W Verheijden JHM The prevalence of isospora-suis and strongyloides-ransomi in suckling piglets in the netherlands Vet Quart 1994 16 203 205 10.1080/01652176.1994.9694449 7740744

[B8] Driesen SJ Carland PG Fahy VA Studies on preweaning piglet diarrhea Aust Vet J 1993 70 259 263 8368968 10.1111/j.1751-0813.1993.tb08044.x

[B9] Lindsay DS Dubey JP Blagburn BL Biology of Isospora spp from humans, nonhuman primates, and domestic animals Clin Microbiol Rev 1997 10 19 34 8993857 10.1128/cmr.10.1.19 PMC172913

[B10] Lindsay DS Blagburn BL Biology of mammalian isospora Parasitol Today 1994 10 214 220 10.1016/0169-4758(94)90116-3 15275450

[B11] Otten A Takla M Daugschies A Rommel M The epizootiology and pathogenic significance of infections with Isospora suis in ten piglet production operations in Nordrhein-Westfalen (in German) Berl Munch Tierarztl Wochenschr 1996 109 220 223 8765537

[B12] Torres A Prevalence survey of Isospora suis in twelve European countries Proceedings of international Pig veterinary society congress 2004 Germany: Hamburg 243

[B13] Meyer C Joachim A Daugschies A Occurrence of Isospora suis in larger piglet production units and on specialized piglet rearing farms Vet Parasitol 1999 82 277 284 10.1016/S0304-4017(99)00027-8 10384903

[B14] Aliaga-Leyton A Webster E Friendship R Dewey C Vilaça K Peregrine AS An observational study on the prevalence and impact of Isospora suis in suckling piglets in southwestern Ontario, and risk factors for shedding oocysts Can Vet J 2011 52 184 188 21532828 PMC3022462

[B15] Niestrath M Takla M Joachim A Daugschies A The role of Isospora suis as a pathogen in conventional piglet production in Germany J Vet Med 2002 B49 176 180 10.1046/j.1439-0450.2002.00459.x PMC7165496 12069269

[B16] Rothman KJ Greenland S Modern epidemiology 1998 2nd Philadelphia: Lippincott-Raven

[B17] Stuart BP Gosser HS Allen CB Bedell DM Coccidiosis in swine - dose and Age response to isospora-suis Can J Comp Med 1982 46 317 320 6889908 PMC1320331

[B18] Koudela B Kucerova G Role of acquired immunity and natural age resistance on course of Isospora suis coccidiosis in nursing piglets Vet Parasitol 1999 82 93 99 10.1016/S0304-4017(99)00009-6 10321581

[B19] Martineau GP del Castillo J Epidemiological, clinical and control investigations on field porcine coccidiosis: clinical, epidemiological and parasitological paradigms? Parasitol Res 2000 86 834 837 10.1007/PL00008509 11068816

[B20] Mundt HC Joachim A Becka M Daugschies A Isospora suis: an experimental model for mammalian intestinal coccidiosis Parasitol Res 2006 98 167 175 10.1007/s00436-005-0030-x 16323027

[B21] Skampardonis V Sotiraki S Kostoulas P Leontides L Effect of toltrazuril treatment in nursing piglets naturally infected with Isospora suis Vet Parasitol 2010 172 46 52 10.1016/j.vetpar.2010.04.020 20471754

[B22] Lenth RV Java applets for power and sample size http://www.stat.uiowa.edu/~rlenth/Power

[B23] Donner A Donald A The statistical analysis of multiple binary measurments J Clin Epidemiol 1988 41 899 905 10.1016/0895-4356(88)90107-2 3183697

[B24] McDermott JJ Schukken YH Shoukri MM Study design and analytic methods for data collected from clusters of animals Prev Vet Med 1994 18 175 191 10.1016/0167-5877(94)90074-4

[B25] Sotiraki S Roepstorff A Murrell KD Nielsen JP Maddox-Hyttel C Boes J Thamsborg SM Reduced farrowing contamination level delays spread of Isospora suis and may prevent clinical coccidiosis Proceedings of international Pig veterinary society congress 2004 Germany: Hamburg 826 827

[B26] Langkjaer M Roepstorff A Survival of Isospora suis oocysts under controlled environmental conditions Vet Parasitol 2008 152 186 193 10.1016/j.vetpar.2008.01.006 18289796

[B27] Dohoo IR Quantitative epidemiology: Progress and challenges Prev Vet Med 2008 86 260 269 10.1016/j.prevetmed.2008.02.012 18394733

[B28] Henriksen SA Christensen JP Demonstration of Isospora suis oocysts in faecal samples Vet Rec 1992 131 443 444 1455595 10.1136/vr.131.19.443

[B29] Daugschies A Bialek R Joachim A Mundt HC Autofluorescence microscopy for the detection of nematode eggs and protozoa, in particular Isospora suis, in swine faeces Parasitol Res 2001 87 409 412 10.1007/s004360100378 11403385

[B30] Xie H McHugo G Sengupta A Clark R Drake R A method for analyzing longitudinal outcomes with many zeros Ment Heal Serv Res 2004 6 239 246 10.1023/b:mhsr.0000044749.39484.1b 15588034

[B31] Liu L Ma JZ Johnson BA A multi-level two-part random effects model, with application to an alcohol-dependence study Stat med 2008 27 3528 3539 10.1002/sim.3205 18219701

[B32] Martin W A structured approach for analysing survey data and making useful causal inferences 1997 Paris: Eighth International Symposium on Veterinary Epidemiology and Economics 31 32

[B33] Mickey RM Greenland S A study of the impact of confounder-selection criteria on effect estimation Am J Epidemiol 1987 126 737 737 10.1093/oxfordjournals.aje.a115101 2910056

[B34] Hosmer DW Lemeshow S Applied logistic regression 1989 Chichester: Wiley

[B35] Henderson R Diggle P Dobson A Joint modelling of longitudinal measurements and event time data Biostatistics 2000 1 465 480 10.1093/biostatistics/1.4.465 12933568

[B36] Lillard LA Panis CWA aML multilevel multiprocess statistical software aML multilevel multiprocess statistical software: version 2.0 2003 Los Angeles: Econ Ware

[B37] Mundt HC Daugschies A Wustenberg S Zimmermann M Studies on the efficacy of toltrazuril, diclazuril and sulphadimidine against artificial infection with Isospora suis in piglets Parasitol Res 2003 90 S160 S162 10.1007/s00436-003-0927-1 12928891

[B38] Driesen SJ Fahy VA Carland PG The Use of toltrazuril for the prevention of coccidiosis in piglets before weaning Aust Vet J 1995 72 139 141 10.1111/j.1751-0813.1995.tb15034.x 7646378

[B39] Scala A Demontis F Varcasia A Pipia AP Poglayen G Ferrari N Genchi M Toltrazuril and sulphonamide treatment against naturally Isospora suis infected suckling piglets: Is there an actual profit? Vet Parasitol 2009 163 362 365 10.1016/j.vetpar.2009.04.028 19457615

[B40] Bach U Kalthoff V Mundt HC Popp A Rinke M Daugschies A Luttge B Parasitological and morphological findings in porcine isosporosis after treatment with symmetrical triazintriones Parasitol Res 2003 91 27 33 10.1007/s00436-003-0828-3 12856173

[B41] Mundt HC Mundt-Wustenberg S Daugschies A Joachim A Efficacy of various anticoccidials against experimental porcine neonatal isosporosis Parasitol Res 2007 100 401 411 10.1007/s00436-006-0314-9 17048000

[B42] KilBride AL Gillman CE Green LE A cross sectional study of the prevalence, risk factors and population attributable fractions for limb and body lesions in lactating sows on commercial farms in England B Vet Res 2009 5 30 10.1186/1746-6148-5-30 PMC2744669 19703273

[B43] Straberg E Daugschies A Control of piglet coccidiosis by chemical disinfection with a cresol-based product (Neopredisan 135–1 (R)) Parasitol Res 2007 101 599 604 10.1007/s00436-007-0521-z 17364163

[B44] Sangster NC Managing parasiticide resistance Vet Parasitol 2001 98 89 109 10.1016/S0304-4017(01)00425-3 11516581

