# Supplementary material for: Factors associated with the occurrence and level of Isospora suis oocyst excretion in nursing piglets of Greek farrow-to-finish herds
Source: BMC Vet Res. 2012 Nov 22;8:228. doi: 10.1186/1746-6148-8-228 (PMC3527311; doi:10.1186/1746-6148-8-228)
Supplement: Additional file 1 — Piglet isosporosis questionnaire. [file 1746-6148-8-228-S1.pdf]

PIGLET ISOSPOROSIS QUESTIONNAIRE

Date of visit: ______________________________

The questionnaire was completed by: ______________________________

Region: ______________________________

Owner's name: ______________________________

Farm's name: ______________________________

Address: ______________________________

Telephone number: ______________________________

The interview was carried out in cooperation with:

the farmer __________

the manager __________

other __________

1. Questions regarding management of farrowing and lactation (sows and piglets)

Question	Answer		Variable	Code	
**1.1.** How many sows are there in the herd?			sows		
**1.2.** How many farrowings per sow per year are achieved?			b/s/y		
**1.3.** How many piglets are born alive per litter on average?			litsize		
**1.4.** What is the mean age of weaning?	days		wean		
**1.5.** Sows are vaccinated against	parvovirus		vaccine	1	
	Aujesky virus			2	
	PRRSV			3	
	E.coli			4	
	Clostridium spp			5	
	Erysipelas			6	
	Leptospira			7	
	Atrophic rhinitis			8	
	other			9	
**1.6.** What kind of vaccine is used against	dead	alive	livedead	0	1
-Aujesky's disease					
-PRRSV disease					
**1.7.** What is the medical scheme for treatment against endoparasites in sows?	product name	frequency	antipar		

2. Questions regarding management of farrowing rooms

Question	Answer		Variable	Code	
**2.1.** How many farrowing rooms are there?	Nr of farrowing rooms		farroom		
**2.2.** Is all-in-all-out practise applied consistently in the farrowing rooms?	YES	NO	inout	1	0
**2.3.** For how many days are farrowing rooms left empty from one batch of sows to another?	days		sowtosow		
**2.4.** How many days before the expected date of farrowing are sows transferred into the farrowing pen?	days		timetofar		
**2.5.** Are all pens in a farrowing room filled at once during transfer of sows?	YES	NO	full	1	0
**2.6.** If not, the empty farrowing pens	remain empty		empty	1	
	are filled in later			2	
**2.7.** Is tranfer of sows continued in a farrowing room after the initiation of farrowings?	YES	NO	enteraffar	1	0
**2.8.** Do you perform cross-fostering of piglets? If so, at what time post farrowing (p.f.)?	NO		foster	0	
	in the first 24h			1	
	after the first 24h			2	
**2.9.** Is there a problem of diarrhoea in nursing piglets?	YES	NO	diarhoea	1	0
**2.10.** If so, how long does this problem exist?			howlong		
**2.11.** If so, in which week of piglets' life does is it manifested?	1st week		weekdiar	1	
	2nd week			2	
	3rd week			3	
	4th week			4	
**2.12.** Has a diagnosis of the cause of diarrhoea been established?	YES	NO	diagn	1	0
**2.13.** If so, which was the causative agent?	E.coli		causediar	1	
	Clostridium spp.			2	
	Isospora suis			3	
	TGE			4	
**2.14.** Are there any specific farrowing pens, where resident litter experience diarrhoea more often?	YES	NO	certpen	1	0
**2.15.** If so, in which site of the farrowing room are they located?	entrance		certpenloc	1	
	windows			2	
	scattered			3	
**2.16.** Is the manifested diarrhoea lifethreatening for piglets?	never		death	0	
	rarely			1	
	often			2	
**2.17.** If so, is any medical treatment applied?	YES	NO	treat	1	0
**2.18.** If so, what is the treatment?	drug	frequency	whattreat		

**2.19.** Do you observe any seasonal effect in the frequency of occurrence of diarrhoea?	YES	NO	seasonincr	1	0
**2.20.** If so, during which season of the year?	spring		season	0	
	summer			1	
	fall			2	
	winter			3	
**2.21.** If so, what is the percentage of litters with diarrhoea per season, approximately?	season	%			
	spring			1	
	summer		seasonper	2	
	fall			3	
	winter			4	
**2.22.** Is there a problem of diarrhoea in weaned piglets?	YES	NO	weaneddiar	1	0
**2.23.** What is the inclusion of soy in the ration during the first 15 days after weaning?			soy		
**2.24.** Is any treatment with antibiotics applied in nursing piglets?	YES	NO	antib	1	0
**2.25.** Do you use any anticoccidial treatment nursing piglets?	YES	NO	anticocuse	1	0
**2.26.** If so, which one?	anticoccidial		anticoc		
**2.27.** How long have you been using it?			usedur		
**2.28.** On which day of piglets' life is the anticoccidial administered?	day p.f.		dayadm		
**2.29.** Is this treatment effective against the observed diarrhea?	YES	NO	effanticoc	1	0

3. Questions regarding properties of farrowing facilities

Question	Answer		Variable	Code	
**3.1.** In how many buildings is farrowing section housed?	nr of buildings		facil		
**3.2.** How many farrowing rooms are there in each building?			farroom		
**3.3.** Are farrownig rooms completely separeted with each other?	YES	NO	separate	1	0
**3.4.** How many farrowing pens are there per room?			penroom		
**3.5.** What kind of material is the flooring of the pen made of?	plastic			1	
	concrete		penkind	2	
	metal			3	
**3.6.** Pen partitions are solid or perforated?	solid	perforated	pensep	1	2
**3.7.** Is direct contact between piglets of neighbouring pens possible?	YES	NO	dircontact	1	0

**3.8.** Are there slatted floors in the farrowing section?	YES	NO	slatfloor	1	0
**3.9.** What is the portion of slatted flooring in the farrowing pen?	100%		slatpart	0	
	60%			1	
	30%			2	
**3.10.** What is the type of flooring in the creep area of the farrowing pen?	perforated	unperforated	restfloor	1	2
**3.11.** If it is not perforated, what kind of material is used?	plastic		restflloormat	1	
	wood			2	
	concrete			3	
	other			4	
**3.12.** What is the type of flooring of the farrowing pen behind the sow?	perforated	unperforated	sowfloor	1	2
**3.13.** Are there any sites in the pen where water or other liquids are accumulated?	YES	NO	liquid	1	0
**3.14.** Is there a mechanichal ventilation system in the farrowing rooms?	YES	NO	ventilation	1	0
**3.15.** If so, does it operates:			ventuse		
a. only during warm months of the year?	a			1	
b. during all year?	b			2	
c. no longer operative?	c			3	
**3.16.** Ventilation system is:			venttype		
a. automatically controlled by temperature & r/h	a			1	
b. automatically controlled by temperature	b			2	
c. manually operated	c			3	
**3.17.** What is the temperature that ventilation is set to operate?	temp (ºC)		temp		
**3.18.** What is the capacity of the ventilation system?	m3/h		capacity		
**3.19.** How many scales of operation does the manualy ventilation system have?			scales		
**3.20.** In what scale does it operate in winter?			winter		
**3.21.** In what scale does it operate in summer?			summer		

4. Questions regarding sanitary measures and hygiene in the farrowing section

Question	Answer		Variable	Code	
**4.1.** Are all litters of a farrowing room weaned simultaneously, before introduction of the next batch of sows?	YES	NO	allweaned	1	0
**4.2.** Is the flooring of the farrowing pen cleaned during lactation period?	YES	NO	cleanlact	1	0
**4.3.** If so, how often is it cleaned?			howoften		
**4.4.** Are farrowing facilities always cleaned before introduction of the next batch of sows?	YES	NO	befclean	1	0
**4.5.** Betwenn batches, do you clean per	pen		cleanempty	1	
	room			2	
	farrowing unit			3	

**4.6.** Which is the way of cleaning?	mechanical removal<br>high pressure water<br>water plus detergent<br>other		cleanway	1<br>2<br>3<br>4	
**4.7.** Washing is performed with hot or cold water?	hot	cold	water	1	2
**4.8.** Are there visible feacal debris after cleaning?	YES	NO	remainfeac	1	0
**4.9.** If there are feacal debris, in which sites of the farrowing pen are located?	floor		locremfeac	1	
	corners			2	
	side partitions			3	
**4.10.** Does cleaning include both flooring and partitions of the pen?	YES	NO	penclean	1	0
**4.11.** Do you disinfect after cleaning?	always		disinf	3	
	often			2	
	rarely			1	
	never			0	
**4.12.** If so, which disinfectant is used?	product name		typedisinf		
**4.13.** What is the duration of downtime (in days)?	days		dry		
**4.14.** Is there any drying material used?	YES	NO	drymat	1	0
**4.15.** If so, which product is used?	product name		whatdrymat		
**4.16.** Are cleaning and disinfection measures applied in all farrowing pens of a room every time?	YES	NO	cleanallpen	1	0
**4.17.** Are these measures applied also in the corridors of the farrowing room?	YES	NO	roomcorr	1	0
**4.18.** Is ther any posssibility of dispersion of dirt in adjacent pens during cleaning?;	YES	NO	spread	1	0
**4.19.** How many caretakers are employed in the farrowing section?			caretakers		
**4.20.** Do caretakers enter farrowing pens during manipulations in piglets?	YES	NO	enterpen	1	0
**4.21.** Is there any roddent control applied?	YES	NO	extermin	1	0
**4.22.** Are there any animals with possible access to the farrowing unit?	YES	NO	otheranim	1	0
**4.23.** If so, what kind of animals?	cats		animals	1	
	dogs			2	
	birds			3	
